# Novel Molecular Approaches to the Understanding of Pathophysiologal Mechanisms Acting in Human Embryos: An Editorial

**DOI:** 10.3390/ijms23084327

**Published:** 2022-04-14

**Authors:** Gabor L. Kovacs, Katalin Gombos

**Affiliations:** 1 National Laboratory on Human Reproduction, University of Pécs, Ifjúság u. 20, 7624 Pécs, Hungary; gombos.katalin@pte.hu; 2 János Szentagothai Research Centre, University of Pécs, Ifjúság u. 20, 7624 Pécs, Hungary; 3 Department of Laboratory Medicine, Medical School, University of Pécs, Ifjúság u.13, 7624 Pécs, Hungary

Infertility is a rapidly evolving global health problem. The number of babies born through in vitro fertilization has reportedly overtaken 8 million people. The global trend is to partiality perform elective single embryo transfers, to avoid risks associated with multiple pregnancies and to improve the probability of live-birth in complete health. There is a growing interest in understanding embryonic pathophysiology, especially during the pre-implantation and peri-implantation period. This Special Issue intends to highlight the shaded line between the normal and pathological molecular behavior of the pre-implantation embryo and those maternal and embryonic molecular markers that can be useful to enhance the effectiveness of in vitro fertilization and predict pregnancy complications. In in vitro fertilization procedures, the critical process is to assess the viability and the pathophysiological risks during culturing. Recently emerging multi-omics approaches have enabled us to take steps toward being less invasive and at the same time to enhance the molecular resolution of the experimental specimen. On this point, the review article by Zmuidinaite et al. [1] contributes to the estimation of the research translation potential into clinically applicable methods that ultimately improve live birth and reduce time to pregnancy. They summarize the current technological progress of reproductive medicine towards the development of quantitative and noninvasive testing tools. They outline the latest research achievements for noninvasive assays: cell-free DNA analysis, microscopy techniques coupled with artificial intelligence and omics analysis of the spent blastocyst media. High-throughput proteomics and metabolomics technologies are also estimated to be promising for noninvasive embryo analysis. The biggest advantages of such technology are that they can differentiate between the embryos that appear morphologically identical and have the potential to identify the ploidy status noninvasively prior to transfer in a fresh cycle or before vitrification for a later frozen embryo transfer. On this point, the publication of Gombos et al. [2] in this Special Issue demonstrates that non-invasive pre-implantation genetic testing for aneuploidy (NIPGT-A) is a potentially appropriate test to assess chromosomal ploidy of the embryo; moreover, the main objective in this study was to provide a comprehensive workflow for a clinically applicable strategy for NIPGT-A. The study methodologically involves next-generation sequencing (NGS) technology. Their retrospective study was performed on spent blastocyst culture media of Day 3 embryos fertilized with intracytoplasmic sperm injection (ICSI) with good quality score on morphology assessment. The authors identified chromosomal abnormalities by an optimized bioinformatics pipeline applying a copy number variation (CNV) detecting algorithm. In this study, Gombos et al. [2] demonstrate a comprehensive workflow covering both wet- and dry-lab procedures supporting a clinically applicable strategy for NIPGT-A (Figure 1). The described integrated approach of non-invasive evaluation of embryonic DNA content of the culture media can potentially supplement existing pre-implantation genetic screening methods.

Early blastocysts are very adaptive to their environment, and embryonic development is a process of complex events orchestrated by the genome and later influenced by the maternal molecular microenvironment. In recent years, scientist have paid close attention to the role of small, non-coding RNAs (sncRNA) in embryo implantation and physiological embryonic development and demonstrated a multifunctional effect on the transcriptional and post-transcriptional levels of gene regulation. Timofeeva et al. [3] identified key sncRNA molecules that participate in maternal-to-zygotic transition and reflect differentiating potential and physiological fetal developmental competence. They carried out sncRNA deep sequencing followed by quantitative real-time RT-PCR analyzed sncRNA profile in spent culture medium from morula with different development potentials: no potential (degradation/developmental arrest), low potential (poor-quality blastocyst), and high potential (good/excellent quality blastocyst capable of implanting and leading to live birth). They have shown that the quality of embryos at the morula stage is determined by secretion/uptake rates of a set of eight different piRNAs in correlation with hsa-let-7b-5p and hsa-let-7i-5p. The predicted gene targets of the sncRNA panel are proved to be decreased at the eight-cell morula–blastocyst stage and critical to early embryo development. As a part of an optimization strategy introduced in the assisted reproductive technology program, Timofeeva et al. [3] obtained original data on sncRNA profiling in spent culture medium. These findings provide an insight into the expansive RNA interference network that controls human embryogenesis at the pre-implantation stage.

Recently, the wide-spread application of time-lapse-monitoring has provided the possibility of continuous observation of the developing embryos during culturing. Despite the morphokinetic analysis, this method failed to elucidate the subject of early embryonic developmental arrest. Sfakianoudis et al. [4]—based on an elegant systematic review—concluded that the developmental arrest of the pre-implantation embryo is a multifactorial condition, characterized by lack of cellular division for at least 24 h. The systematic review, including 76 studies, collects and discusses all available data on the molecular drivers of developmental arrest, focusing on both the embryonic and the parental factors. The identified embryonic factors associated with arrest included gene variations, mitochondrial DNA copy number, methylation patterns, chromosomal abnormalities, metabolic profile and morphological features. Parental factors included gene variation, protein expression levels and infertility etiology. A valuable conclusion emerging through critical analysis indicated that genetic origins of developmental arrest analyzed from the perspective of parental infertility etiology and the embryo itself share common ground. This is a unique and long-overdue contribution to literature that, for the first time, presents an all-inclusive methodological report on the molecular drivers leading to pre-implantation embryos’ arrested development. This systematic review provides the basis of a complex assessment strategy to improve in vitro fertilization cycle outcome.

In a comprehensive review, Ali et al. [5] deal with pregnancy complications that are a major cause of fetal and maternal morbidity and mortality in humans. They outline that the majority of pregnancy complications initiate due to abnormal placental development and function. They evaluate the efficacy of miRNAs as potential biomarkers expressed in placenta and appeared in the maternal circulation to predict pregnancy complications. The differential expression of a miRNA in the placenta may not always be reflected in maternal circulation, which makes it difficult to find a reliable biomarker for placental dysfunction. Dysregulation of miRNAs in the placenta not only affects placental development and function, but these miRNAs can also be exported to both maternal and fetal compartments and affect maternal physiology and fetal growth and development. In this review, the authors provide an overview of differentially expressed miRNAs in the placenta and/or maternal circulation during preeclampsia (PE) and intrauterine growth restriction (IUGR), which can potentially serve as biomarkers for prediction or diagnosis of pregnancy complications. Using different bioinformatics tools, Ali et al. [5] also identified potential target genes of miRNAs associated with PE and IUGR and the role of miRNA–mRNA networks in the regulation of important signaling pathways and biological processes.

In conclusion, the manuscripts published in this Special Issue focus on recent—preferably non-invasive—techniques, including time-lapse microscopy, biochemical markers, novel methods of evaluating embryo genomic profile, metabolism and mitochondrial DNA. These tools may identify novel molecular mechanisms, increasing the positive outcome of embryo transfer. Novel technical possibilities undoubtfully give us the opportunity for a deeper insight of the early stage of embryonal life.

## Figures and Tables

**Figure 1:**
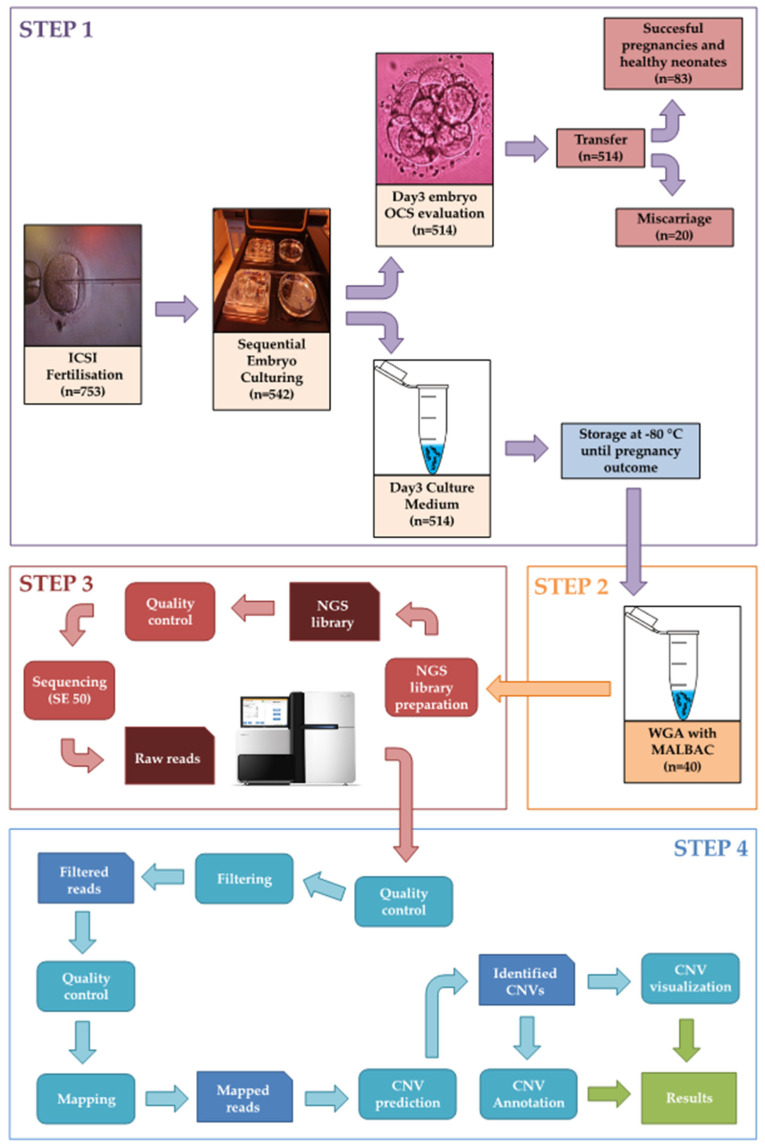
Representation of the NGS-based NIPGT-A workflow including four main steps. Step 1: IVF procedure and sample collection; Step 2: Whole genome amplification; Step 3: Next generation sequencing; Step 4: Bioinformatics analysis. (OCS: “optimized criteria system”, WGA: whole genome amplification, MALBAC: multiple annealing and looping-based amplification).
